# Cerebral Microbleeds and Structural White Matter Integrity in Patients With Traumatic Brain Injury—A Diffusion Tensor Imaging Study

**DOI:** 10.3389/fneur.2022.888815

**Published:** 2022-05-31

**Authors:** Juho Dahl, Olli Tenovuo, Jussi P. Posti, Jussi Hirvonen, Ari J. Katila, Janek Frantzén, Henna-Riikka Maanpää, Riikka Takala, Eliisa Löyttyniemi, Jussi Tallus, Virginia Newcombe, David K. Menon, Peter J. Hutchinson, Mehrbod Mohammadian

**Affiliations:** ^1^Turku Brain Injury Center, Turku University Hospital, University of Turku, Turku, Finland; ^2^Neurocenter, Department of Neurosurgery, Turku Brain Injury Center, Turku University Hospital, University of Turku, Turku, Finland; ^3^Department of Diagnostic Radiology, Turku University Hospital, University of Turku, Turku, Finland; ^4^Perioperative Services, Intensive Care Medicine and Pain Management, Department of Anesthesiology and Intensive Care, Turku University Hospital, University of Turku, Turku, Finland; ^5^Department of Biostatistics, University of Turku, Turku, Finland; ^6^Division of Anaesthesia, Addenbrooke's Hospital, University of Cambridge, Cambridge, United Kingdom; ^7^Neurosurgery Unit, Department of Clinical Neurosciences, Addenbrooke's Hospital, University of Cambridge, Cambridge, United Kingdom

**Keywords:** traumatic brain injury, white matter, cerebral microbleeds, diffuse axonal injury, diffusion tensor imaging

## Abstract

Diffuse axonal injury (DAI) is a common neuropathological manifestation of traumatic brain injury (TBI), presenting as traumatic alterations in the cerebral white matter (WM) microstructure and often leading to long-term neurocognitive impairment. These WM alterations can be assessed using diffusion tensor imaging (DTI). Cerebral microbleeds (CMBs) are a common finding on head imaging in TBI and are often considered a visible sign of DAI, although they represent diffuse vascular injury. It is poorly known how they associate with long-term white matter integrity. This study included 20 patients with TBI and CMBs, 34 patients with TBI without CMBs, and 11 controls with orthopedic injuries. DTI was used to assess microstructural WM alterations. CMBs were detected using susceptibility-weighted imaging (SWI) and graded according to their location in the WM and total lesion load was counted. Patients underwent SWI within 2 months after injury. DTI and clinical outcome assessment were performed at an average of eight months after injury. Outcome was assessed using the extended Glasgow Outcome Scale (GOSe). The Glasgow Coma Scale (GCS) and length of post-traumatic amnesia (PTA) were used to assess clinical severity of the injury. We found that CMB grading and total lesion load were negatively associated with fractional anisotropy (FA) and positively associated with mean diffusivity (MD). Patients with TBI and CMBs had decreased FA and increased MD compared with patients with TBI without CMBs. CMBs were also associated with worse clinical outcome. When adjusting for the clinical severity of the injury, none of the mentioned associations were found. Thus, the difference in FA and MD is explained by patients with TBI and CMBs having more severe injuries. Our results suggest that CMBs are not associated with greater WM alterations when adjusting for the clinical severity of TBI. Thus, CMBs and WM alterations may not be strongly associated pathologies in TBI.

## Introduction

Traumatic brain injury (TBI) is the leading cause of mortality among young adults (1). In TBI, momentary shear forces can trigger a pathophysiological process that continues several months after injury. This process known as diffuse axonal injury (DAI) leads to long-term deficits in cerebral connectivity and is one of the most common neuropathologic consequences of TBI across all severities (2). As this injury mechanism is not random and diffusely distributed but has specific sites of predilection, the term multifocal axonal injury has also been used.

Diffusion-weighted magnetic resonance imaging (DW-MRI) and—-in particular—-diffusion tensor imaging (DTI) can provide rich information on white matter (WM) microstructure and structural connectivity of the brain after TBI (1, 3, 4). This is achieved by measuring the rate and directionality of water diffusion. Subtle changes in the diffusivity are associated with disruptions in the integrity of the WM. Several scalar metrics characterizing the diffusion can be derived for each voxel in DTI (5). In TBI, disruptions in the WM tracts are usually represented as decreased fractional anisotropy (FA), or in other words, loss of asymmetry of diffusion (6). The total amount of diffusion may also be affected, which can be seen as increased mean diffusivity (MD). These changes associate with impaired cognitive performance (7). FA has been shown to be a reproducible and relatively sensitive measure to detect WM disruptions in TBI (8). The most common analysis methods for DTI data include region-of-interest (ROI) and voxel-wise approaches. One of the most commonly implemented voxel-wise methods is tract-based spatial statistics (TBSS), in which the analysis is restricted to a FA skeleton to reduce registration errors (9, 10).

Cerebral microbleeds (CMBs) are very common, yet relatively poorly studied parenchymal findings following TBI. CMBs associated with TBI represent extravasation of blood from small adventitial vessels ruptured by shear forces—-making them a distinct pathophysiological entity from primary injury to the axons (11, 12). Susceptibility-weighted imaging (SWI) is a standard method for detecting CMBs after TBI (13, 14). SWI is able to detect significantly more CMBs than fluid-attenuated inversion recovery and twice as many as gradient recalled echo (13, 14). Detection of CMBs using SWI in early diagnostics of TBI is considered a marker of the severity of the injury (15). Total CMB volume has also been found to associate with worse outcome in adults (13, 14, 16). However, detection of CMBs or other lesions are not sensitive for DAI and often underestimate the injury (17). Significant long-term disability after TBI can occur without CMBs or other significant lesions and it remains scarcely studied whether CMBs associate with long-term structural WM integrity (18–20). Previous evidence suggests that CMBs and WM integrity are not necessarily closely related (21, 22).

The focus of this study was to further examine the relationship between the presence of CMBs and structural WM integrity in TBI. We also examined the relationship of CMBs with long-term functional clinical outcome. This may give novel insight to refocusing imaging strategies for TBI.

## Materials and Methods

This study was based on data collected during the EU-funded International Evidence-based Diagnostic and Treatment Planning Solution for Traumatic Brain Injuries (TBIcare) project. From 620 patients with acute head injuries, a total of 203 patients with TBI and 40 patients with orthopedic injuries were recruited during the TBIcare project at Turku University Hospital, Turku, Finland, between years 2011 and 2013. All participants gave written informed consent.

The ethical review board of the Hospital District of Southwest Finland has approved the study protocol (decision 68/180/2011).

### Inclusion Criteria

The inclusion criteria for the original TBIcare project were age ≥ 16 years, clinical diagnosis of TBI, and indication for acute head CT scan according to National Institute for Health and Care Excellence criteria (available at http://www.nice.org.uk/guidance/cg176). Exclusion criteria were age <16 years at study entry, blast-induced or penetrating injury, chronic subdural hematoma, inability to live independently because of pre-existing brain disease, TBI or suspected TBI not needing head CT scan, > 2 weeks from the injury, nor living in the district and thus preventing follow-up visits (Turku), not speaking a native language, and no consent received (23).

Additional inclusion criteria for this sub-study were availability of education level data, availability of DTI imaging data between 2 months and 2 years after injury, and availability of data on the clinical TBI severity including GCS and PTA. In addition, we applied further exclusion criteria to avoid confounders for the WM/CMB analyses as follows:

- Age > 50 years (to reduce the number of age-related WM abnormalities)
- Focal intracerebral lesions over 5 cm^3^ (estimated visually by a neuroradiologist, to enable proper WM mapping)
- Severe hypertension (>180/120 mmHg) (24) (to reduce the risk of including non-traumatic CMBs)

**Figure 1:**
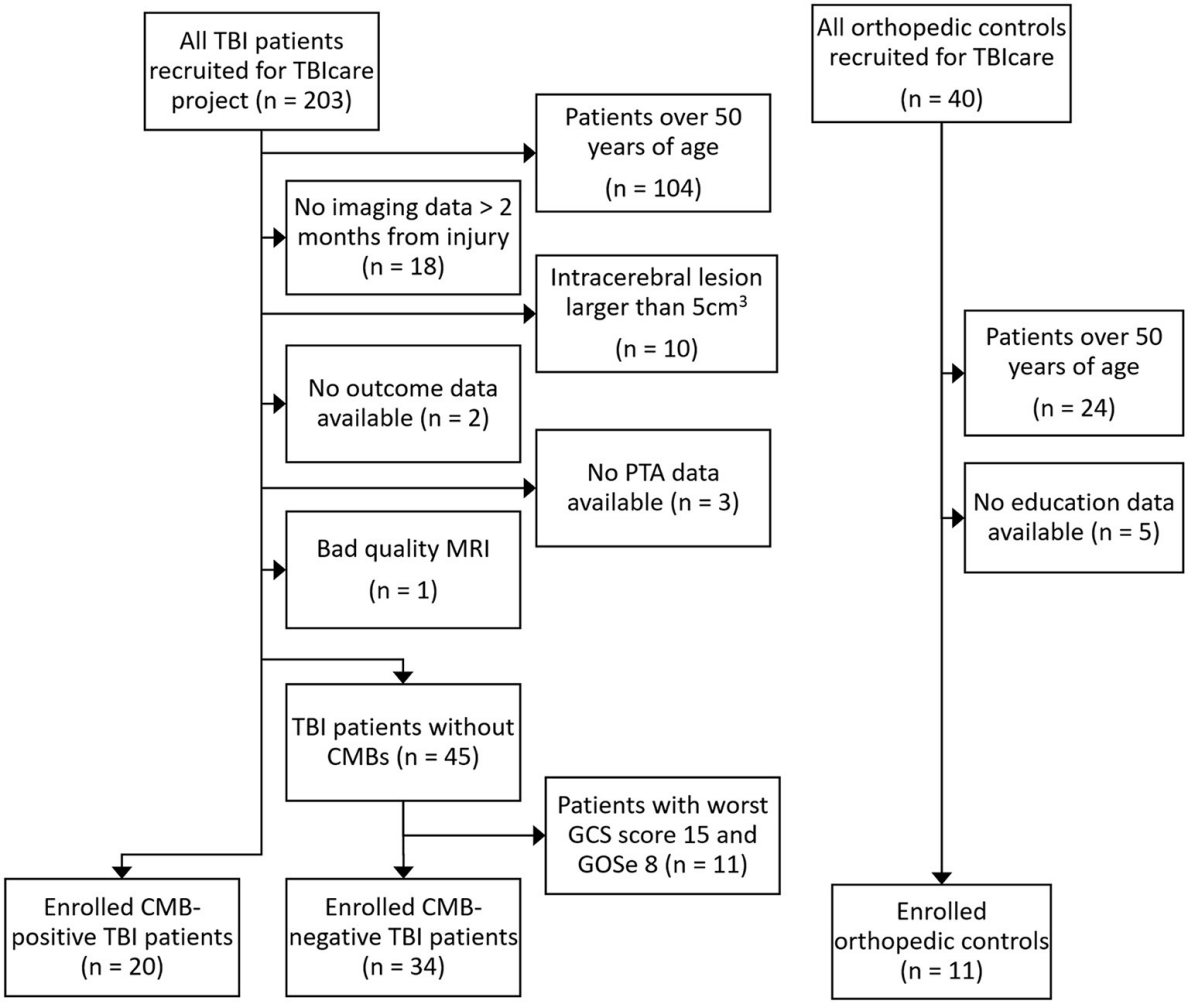
Flowchart of inclusion and exclusion of the study groups. TBIcare, EU-funded international Evidence-based Diagnostic and Treatment Planning Solution for Traumatic Brain Injuries; TBI, traumatic brain injury; PTA, post-traumatic amnesia; MRI, magnetic resonance imaging; CMB, cerebral microbleed, GCS, Glasgow Coma Scale, GOSe, Glasgow Outcome Scale extended.

We also excluded patients with TBI with no CMBs whose lowest GCS was 15 and who had full recovery (GOSe 8) in order to improve the comparability of the groups as patients with CMBs had clinically more severe injuries. The formation of the study population is shown as a flowchart in Figure 1.

### Clinical Measures

To measure the clinical severity of TBI, GCS score (from the scene of accident and the emergency department) and PTA were used. The prehospital GCS was available for all included patients. Emergency department GCS data were available for 51 out of 54 patients with TBI. The lowest recorded GCS score for each subject was used for statistical analysis. PTA was assessed retrospectively by a clinical neurologist during a routine follow-up visit and measured in three categories: <24 h, 1–7 days, and > 7 days. Outcome was assessed at a median of 252 days after injury using the Glasgow Outcome Scale extended (GOSe) ranging from 1 (dead) to 8 (upper good recovery) (25). Other subject characteristics included in statistical analyses were age at the time of injury, sex, and education level measured on a 4-point scale: basic school, lower level professional, upper level professional, and academic education. Demographics of the subjects are presented in Table 1.

**Table 1:** Demographics and characteristics of the patients.

		**All patients with TBI** **(*n* = 54)**	**TBI, CMB present** **(*n* = 20)**	**TBI, CMB absent** **(*n* = 34)**	**Controls** **(*n* = 11)**	***P*–value**
Age, median (range), years		31 (18–50)	24 (19–47)	32 (18–50)	29 (22–49)	0.32 ^a^
Sex, female count (%)		16 (30)	3 (15)	13 (38)	4 (36)	0.18 ^b^
Education, count (% of group)	*basic school*	18 (33)	8 (40)	10 (29)	0	0.070 ^b^
	*lower–level professional*	22 (41)	5 (25)	17 (50)	8 (73)	
	*upper–level professional*	12 (22)	6 (30)	6 (18)	3 (27)	
	*academic education*	2 (4)	1 (5)	1 (3)	0	
GCS score, median (range)		15 (3–15)	15 (3–15)	15 (11–15)	N/A	0.016 ^c^
GCS score, count (% of group)	*13–15*	44 (81)	12 (60)	32 (94)	N/A	
	*9–12*	4 (7)	2 (10)	2 (6)		
	*3–8*	6 (11)	6 (30)	0		
Post–traumatic amnesia	* <1 day*	35 (65)	8 (40)	27 (79)	N/A	0.002 ^c^
count (% of group)	*1–7 days*	6 (11)	2 (10)	4 (12)		
	*>7 days*	13 (24)	10 (50)	3 (9)		
GOSe score, median (range)		7 (3–8)	7 (3–8)	7 (5–8)	N/A	0.76 ^c^
Injury mechanism	*Traffic accident*	26 (48)	12 (60)	14 (41)	N/A	0.13 ^b^
count (% of group)	*Accidental fall*	20 (37)	8 (40)	12 (35)		
	*Assault*	**7** (13)		7 (21)		
	*Horse kick*	1 (2)		1 (3)		
Days from injury to SWI, median (range)		17 (1–56)	22 (1–56)	16 (1–50)	N/A	0.78 ^d^
Days from injury to DTI, median (range)		243 (178–429)	236 (186–413)	246 (178–429)	28 (8–43)	0.73 ^d*^
Days from injury to outcome assessment, median (range)		252 (178–556)	248 (186–470)	260 (178–556)	N/A	0.95 ^d^

P–values are presented for a three–way comparison between the two groups of patients with TBI and controls unless marked with an asterisk. ^*^Only compared within patients with TBI; a, Kruskal–Wallis test, b, Fisher's exact test, c, Ordinal logistic regression, d, Mann–Whitney U test, CMB, cerebral microbleed, GCS, Glasgow Coma Scale, GOSe, Glasgow Outcome Scale extended, SWI, susceptibility–weighted imaging, DTI, diffusion tensor imaging.

### MRI Acquisition and Visual Evaluation

For SWI, the first available MRI session was used. For DTI, the first available session between two months and 2 years after injury was used. More detailed information on MRI timing is shown in Table 1.

MR imaging was performed with a 3T MRI scanner (Siemens Magnetom Verio, Siemens Healthcare, Erlangen, Germany). DW-MRI was performed using a spin-echo echo-planar imaging sequence with a repetition time of 11.7 s, echo time 106 ms, 2 × 2 × 2 mm voxel size resulting in 77 axial slices and a field of view of 192 × 192 mm. Sixty-four encoding directions uniformly distributed on a unit sphere with diffusion gradients of b = 1000 s/mm^2^ were used. SWI was obtained with a repetition time of 28 ms, echo time 20 ms and 0.6 × 0.5 × 1.2 mm voxel size. Additional acquired MRI sequences included magnetization prepared rapid gradient echo, proton density weighted, T2-weighted, and gradient recalled echo.

A radiological grading system (later referred to as CMB grading) was used as the main variable categorizing CMBs according to their anatomical location (26). In this grading system, patients with only hemispheric lesions were classified into CMB grade I, patients with lesions in corpus callosum into CMB grade II, patients with lesions in the brainstem into CMB grade III and patients with lesions in substantia nigra or mesencephalic tegmentum into CMB grade IV. No CMBs was included as an additional CMB grade 0, resulting in a total of 5 categories. The total number of CMBs (later referred to as total lesion load) was also counted and used for statistical analysis. Visual evaluation of MR images was carried out by an expert neuroradiologist. The grading system has been shown to have a very good inter-rater agreement (26).

### DW-MRI Analysis

DW-MR images were corrected for eddy current distortions, subject motion, and susceptibility distortions and fitted for diffusion tensors using ExploreDTI software (27). FA and MD (later referred to as DTI metrics) images were calculated from the pre-processed DW-MRI data for each subject. All DW-MRI analyses in patients with TBI were performed both with and without adjusting for GCS and PTA as covariates unless specified otherwise. This was done to investigate the predictive potential of CMBs on white matter microstructure independent of clinical severity.

#### Tract-Based Spatial Statistics

TBSS approach in Oxford Center for Functional MRI of the Brain Software Library (FSL) was used to analyze the images (9, 28). First, a non-linear registration was used to align all individual FA images into MNI 152 standard space. A mean FA image of all subjects was then calculated, and a mean FA skeleton was created. All individual subjects' FA images were then projected onto the mean FA skeleton. In addition to FA, the original non-linear registration and projection onto the mean FA skeleton was applied for MD. Voxel-wise linear correlation analyses were conducted between DTI metrics and CMB variables (CMB grading and total lesion load) within patients with TBI using the *randomize* tool with 5,000 random permutation tests (29). Voxel-wise DTI metrics were further analyzed in three individual group comparisons between all three patient groups using *randomize*. Despite the limited effect of age, sex, and education on DTI metrics in the whole brain analysis, they were included in the linear model as covariates in all TBSS analyses as they have been shown to affect DTI metrics and may be significant in a voxel-wise approach (30, 31). Time from injury to DTI was also included as a covariate for TBSS analyses within patients with TBI as DTI metrics are shown to change in the chronic phase of TBI (4). Threshold-free cluster enhancement was used to enhance cluster-like structures (32). The resulting *P*-value images were corrected for multiple comparisons across space.

#### Whole Brain Analysis

In addition to the TBSS analyses, whole brain analysis was performed to evaluate the association between CMB variables and DTI metrics, and the differences in DTI metrics between the patient groups (8). In the whole brain analysis, a scalar mean value was calculated from skeletonized FA and MD images for each subject. Linear models were built to examine the association between DTI metrics and CMB grading in the presence of relevant background factors (age, sex, education, time from injury to DTI) as well as assessments for clinical severity of injury (GCS, PTA). Naturally only patients with TBI could be included in these models. First, a univariate model was tested and while all background factors were non-significant, the final model included CMB grading, PTA, and GCS. In addition to CMB grading, the same procedure was repeated for total lesion load and for a group comparison between the two groups of patients with TBI. Whole brain DTI metrics were compared between controls and the two groups of patients with TBI individually in univariate linear models.

The associations between mean whole brain DTI metrics and outcome (GOSe) were assessed using partial Spearman's correlation covariate-adjusted for relevant background characteristics (age, sex, education, time from injury to DTI) and clinical severity (GCS, PTA) within all patients with TBI as well as within the two groups of patients with TBI independently.

#### Region of Interest Analysis

For an additional ROI analysis, mean FA and MD values were calculated for each subject in 48 distinct anatomical WM regions according to the JHU ICBM-DTI-81 WM atlas (33). Regional FA and MD values were then compared between the three groups of patients, and a correlative analysis was conducted between regional mean DTI metrics and CMB variables in a linear model in the same manner as in the whole brain analysis. For ROI analysis, all relevant background factors (age, sex, education, time from injury to DTI) were included as covariates as even though they were non-significant in the whole brain analysis, they might play a role in a more anatomically specific review. Resulting *P*-values were corrected for multiple comparisons using false discovery rate for the 48 WM regions (34).

### Association Between Cerebral Microbleeds and Clinical Outcome

Ordinal logistic regressions were built to analyze the association between CMB variables and clinical outcome. In addition to the univariate models, covariate-adjusted models including the GCS were built to estimate the association between CMBs and outcome independent of clinical severity. As ordinal logistic regression assumes no multicollinearity, only GCS was used as the measure of clinical severity. Groupwise testing for the effect of CMBs on outcome is presented in Table 1.

### Other Statistical Analyses

Groupwise testing for background characteristics is shown in Table 1. For the parametric analyses, normality of the data was checked visually as well as by using the Kolmogorov-Smirnov test. Equality of variance was tested for all with the Student's *t*-tests using Bartlett test (35). The proportional odds assumption for ordinal logistic regressions was tested using the Brant test (36). All statistical analyses were conducted using Rstudio (version 1.1.456), SAS software, Version 9.4 of the SAS System for Windows (SAS Institute Inc., Cary, NC, USA) or FSL as mentioned in the DW-MRI analysis sub-section. All results with *P*-values below 0.05 (two-tailed) were considered statistically significant.

## Results

### Subjects

The final population for this study consisted of 54 patients with TBI, of which 20 had CMBs, and 11 patients with orthopedic injuries who served as controls.

### Clinical Characteristics

As shown in Table 1, most of the TBIs were mild based on either the GCS (81 % of all, GCS 13–15 was considered mild) or the duration of PTA (65 % of all, PTA <24 h was considered mild). Patients with TBI and CMBs presented with lower GCS scores (*P* = 0.016) and longer PTAs (*P* = 0.002) compared with patients with TBI without CMBs, although the CMB-negative patients with the mildest injuries were excluded from the study. There were no statistically significant differences between all three groups in age, sex, or education level or time from injury to SWI scan. No significant difference was found between the two groups of patients with TBI in terms of time from injury to DTI, outcome or injury mechanisms. The number of patients in each category of the CMB grading was as follows: grade 0: *n* = 34, grade I: *n* = 9, grade II: *n* = 6, grade III: *n* = 2, grade IV: n = 3.

### DW-MRI Analysis

#### Tract-Based Spatial Statistics

**Figure 2:**
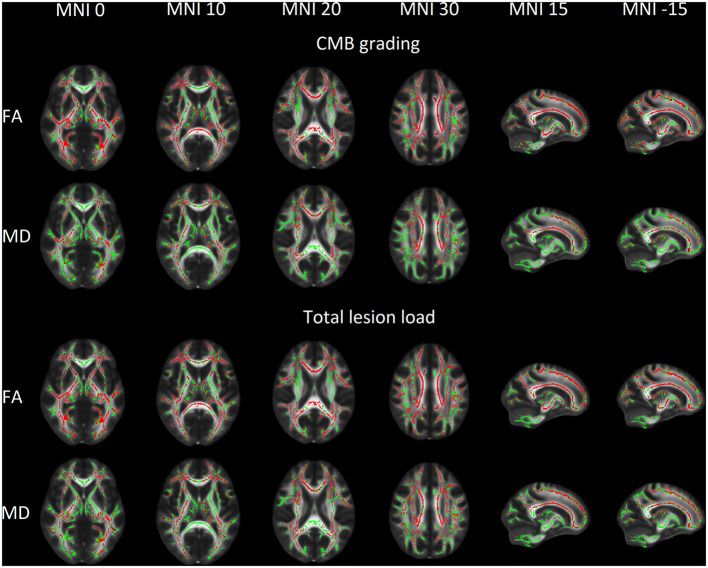
Voxels with a significant linear association between cerebral microbleeds (CMB) and white matter microstructural properties shown in red overlaid on mean fractional anisotropy (FA) skeleton (shown in green) and Oxford Center for Functional MRI of the Brain mean FA template in Montreal Neurological Institute 152 (MNI) space. CMBs show a positive association with FA and a negative association with MD.

TBSS showed that FA correlated negatively with both CMB grading (*P* = 0.0002, *P*-value presented for the most significant voxel) and total lesion load (*P* = 0.0002). MD was positively correlated with both CMB grading (*P* = 0.0006) and total lesion load (*P* = 0.0006). When GCS and PTA were used to control for clinical severity of TBI, no significant correlation between DTI metrics and CMB grading was seen. TBSS results for CMB grading and total lesion load are visually presented in Figure 2.

**Figure 3:**
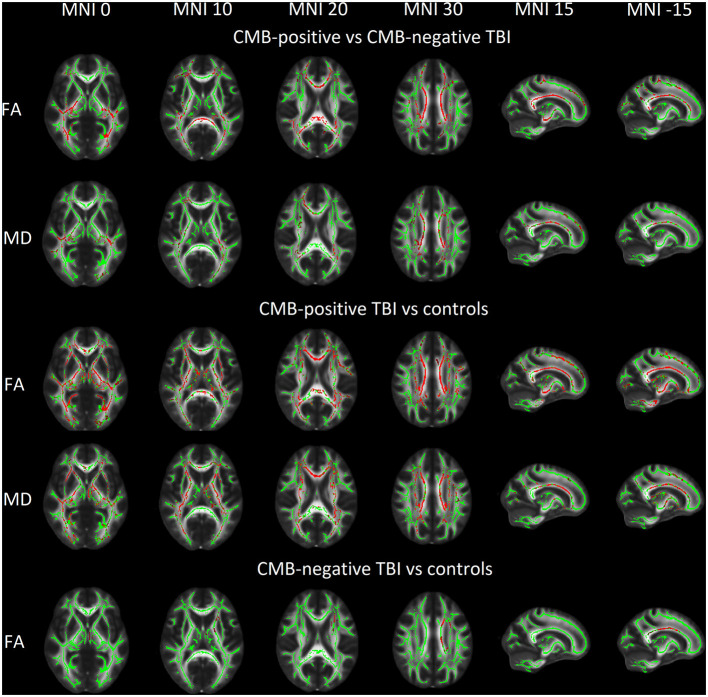
Group comparison of fractional anisotropy (FA) and mean diffusivity (MD) with statistically significant voxels (shown in red) overlaid on mean FA skeleton (shown in green) and Oxford Center for Functional MRI of the Brain mean FA template in Montreal Neurological Institute (MNI) 152 space. CMB, cerebral microbleed, TBI, traumatic brain injury.

Patients with TBI and CMBs presented with lower FA (*P* = 0.007) and higher MD values (*P* = 0.026) compared with patients with TBI without CMBs. When GCS and PTA were additionally used to adjust for clinical severity of TBI, no difference in DTI metrics was found between the two groups of patients with TBI. Patients with TBI and CMBs presented with lower FA (*P* = 0.0008) and higher MD (*P* = 0.004) compared with orthopedic controls, whereas patients with TBI without CMBs had only lower FA compared with orthopedic controls. TBSS results for group comparisons are visually presented in Figure 3.

#### Whole Brain Analysis

**Figure 4:**
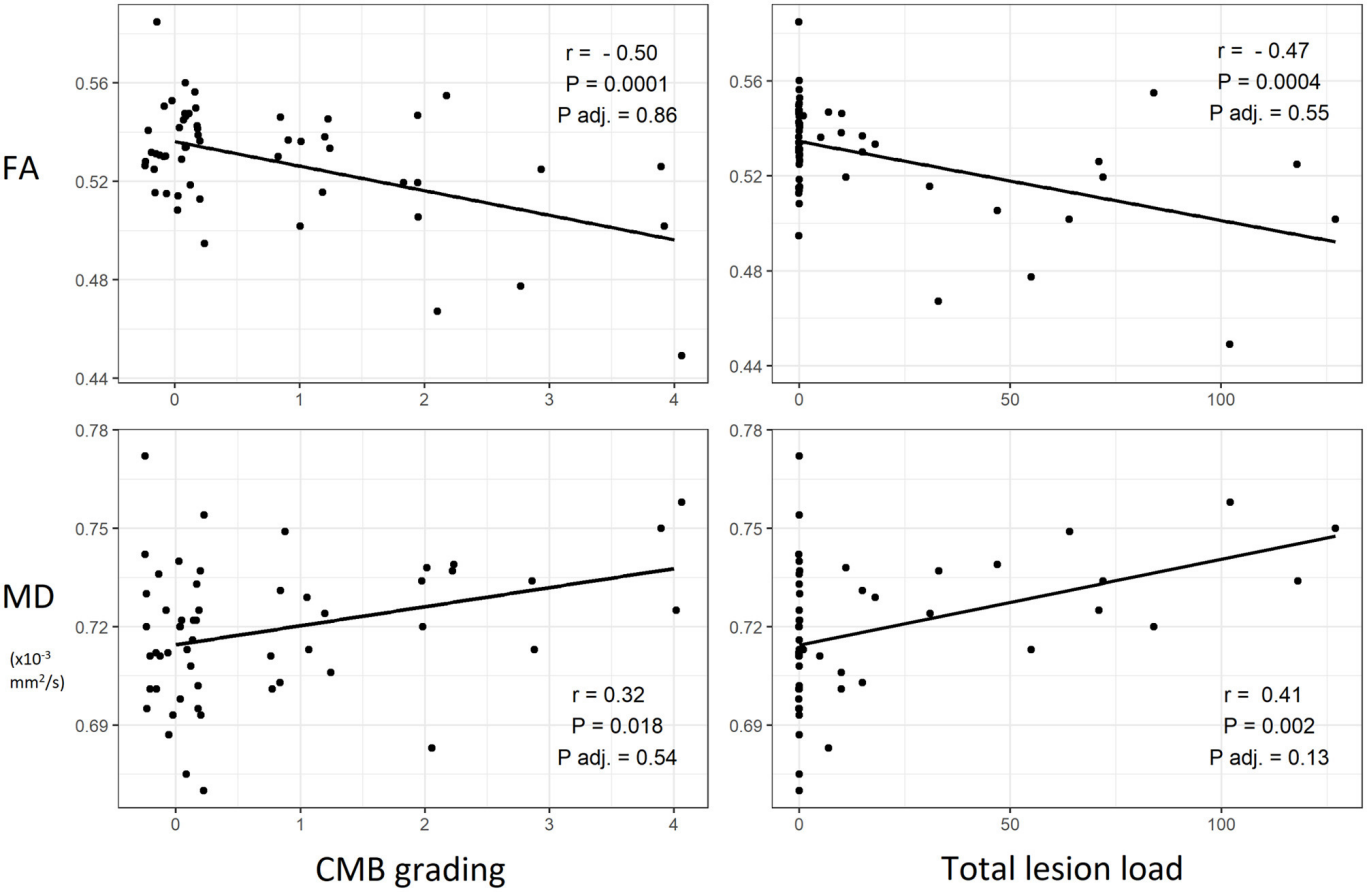
Scatterplots of cerebral microbleeds (CMB) and white matter microstructural properties with best fit lines and corresponding Pearson's correlation coefficients and *P*-values. An additional *P*-value (*P* adj.) for a linear model adjusted for clinical severity of traumatic brain injury (Glasgow Coma Scale and length of post-traumatic amnesia) is shown. FA, fractional anisotropy, MD, mean diffusivity.

CMB grading was negatively correlated with mean whole brain FA (*P* = 0.0001) and positively correlated with mean whole brain MD (*P* = 0.018). Total lesion load was negatively correlated with FA (*P* = 0.0004) and positively correlated with MD (*P* = 0.002). When adjusting for clinical severity, no significant correlation between CMB variables and whole brain DTI metrics was found. Associations between CMB variables and whole brain DTI metrics are visually presented in Figure 4.

Patients with TBI and CMBs presented with lower mean whole brain FA (mean = 0.519, sd = 0.028) compared with patients with TBI without CMBs (mean = 0.535, sd = 0.017, *P* = 0.011). When additionally adjusted for GCS and PTA to account for clinical severity, no significant difference was observed. Patients with TBI and CMBs had higher mean whole brain MD (mean = 0.725 × 10^−3^ mm^2^/s, sd = 0.019 × 10^−3^ mm^2^/s) compared with patients with TBI without CMBs (mean = 0.715 × 10^−3^ mm^2^/s, sd = 0.021 × 10^−3^ mm^2^/s), but the difference was not statistically significant whether adjusting for GCS and PTA or not.

**Figure 5:**
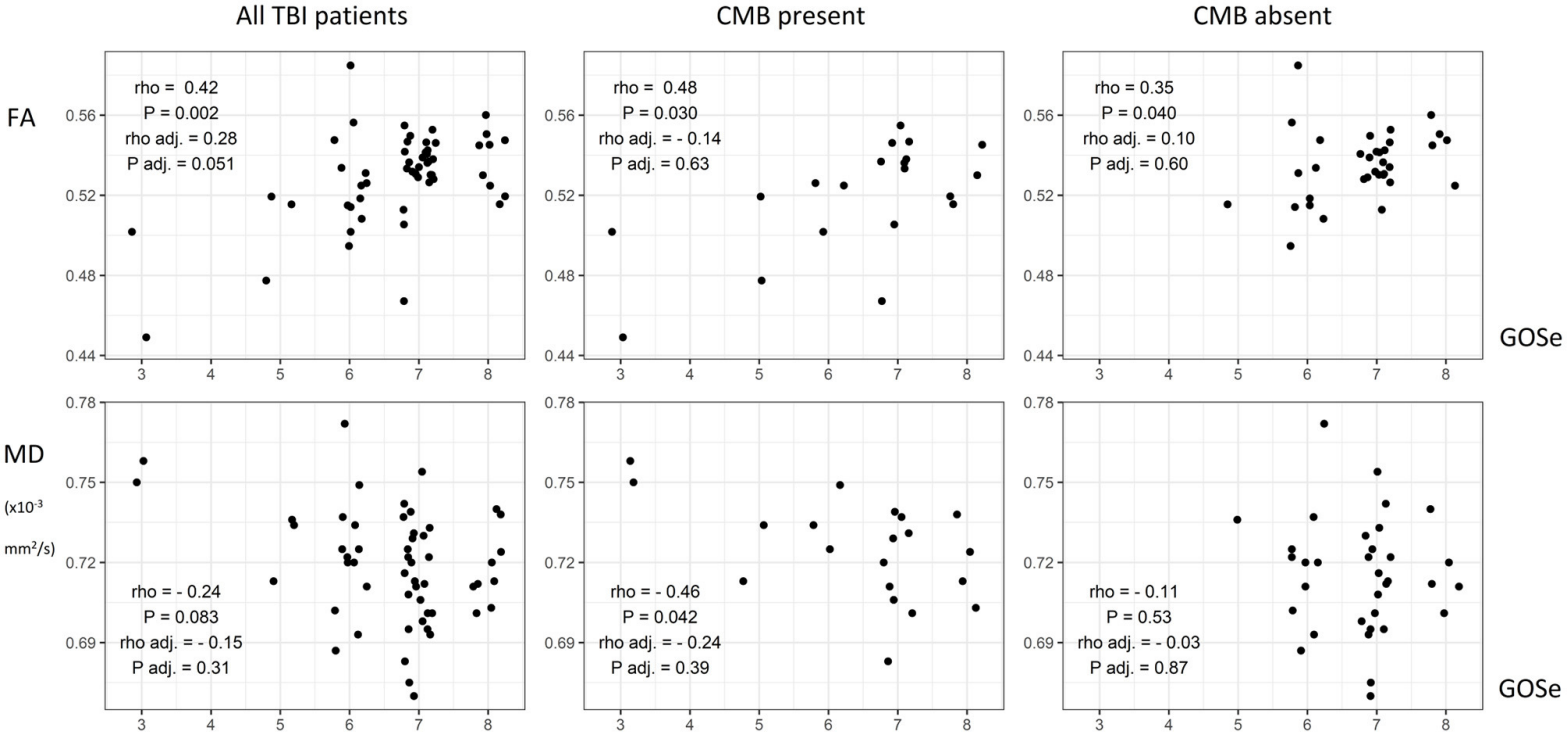
Scatterplots of white matter microstructural properties and outcome including corresponding Spearman's correlation coefficients with *P*-values. Additional coefficients and *P*-values for partial Spearman's correlations adjusted for relevant background factors (age, sex, education level, time from injury to DTI) and clinical severity (Glasgow Coma Scale and length of post-traumatic amnesia) are shown. TBI, traumatic brain injury; CMB, cerebral microbleed; FA, fractional anisotropy; MD, mean diffusivity; GOSe, Glasgow Outcome Scale extended.

When adjusting for relevant background characteristics and clinical severity, no clear statistically significant association between DTI metrics and outcome was found. Associations between whole brain mean DTI metrics and outcome are visually presented in Figure 5.

Of all relevant background factors possibly affecting DTI metrics (age, sex, education, time from injury to DTI), age correlated with FA (r = −0.38, *P* = 0.028) only in patients with TBI without CMBs. Female patients with TBI without CMBs had higher FA (mean = 0.545, sd = 0.015) compared with their male counterparts (mean = 0.529, sd = 0.016, *P* = 0.005). Female patients with TBI and CMBs had higher MD (mean = 0.744 × 10^−3^ mm^2^/s, sd = 0.017 × 10^−3^ mm^2^/s) than their male counterparts (mean = 0.721 × 10^−3^ mm^2^/s, sd = 0.017 × 10^−3^ mm^2^/s, *P* = 0.049). These correlations proved to be non-significant for the comparison between CMB variables and DTI metrics in the whole brain analysis and thus were omitted from the final model.

**Figure 6:**
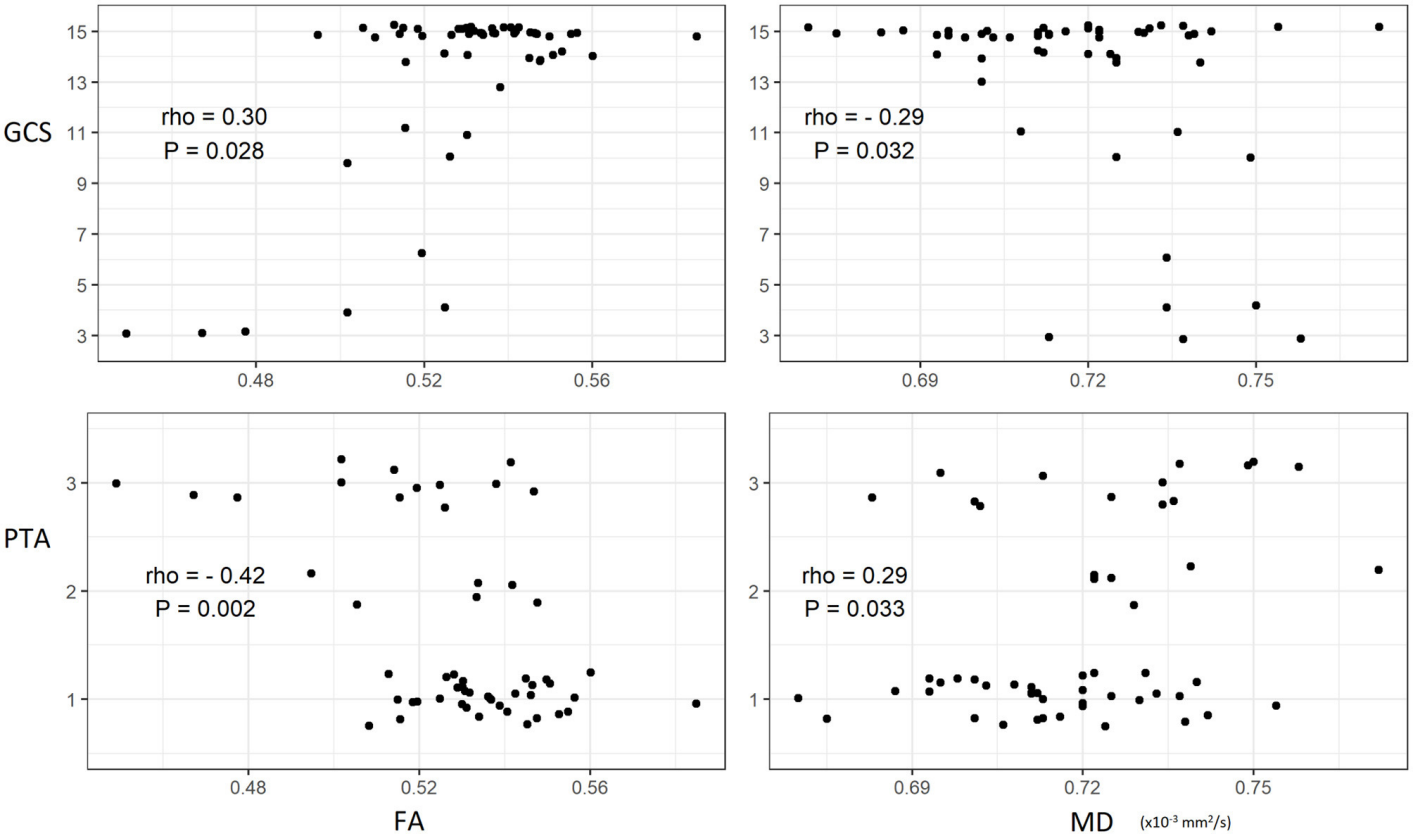
Scatterplots of clinical severity of traumatic brain injury and white matter microstructural properties including best fit lines with corresponding Spearman's correlation coefficients and *P*-values. GCS, Glasgow Coma Scale; PTA, post-traumatic amnesia; FA, fractional anisotropy; MD, mean diffusivity.

Both GCS and PTA correlated with the mean whole brain FA and MD, as illustrated in Figure 6. Similar correlations between clinical severity and DTI metrics were also seen when analyzing the two groups of patients with TBI individually, except for the correlation between PTA and MD.

#### Region of Interest Analysis

In the ROI analysis, CMB grading was negatively correlated with FA in 44 out of all 48 regions and positively correlated with MD in 28 regions. Total lesion load was negatively correlated with FA in 41 regions and positively correlated with MD in 34 regions. When adjusting for GCS and PTA, no significant correlations were found.

No significant difference was found in the ROI analysis between the CMB-positive and CMB-negative patients with TBI whether adjusting for clinical severity or not. Patients with TBI and CMBs presented with lower FA in 28 regions, higher MD in 23 regions and lower MD in 1 region (middle cerebellar peduncle) compared with orthopedic controls. Patients with TBI without CMBs presented with lower FA in 1 region and lower MD in 2 regions (left corticospinal tract and middle cerebellar peduncle) compared with orthopedic controls. The results of the ROI analyses are illustrated in detail in Supplementary Material.

### Association of Cerebral Microbleeds and Clinical Outcome

Higher CMB grade and total lesion load were associated with a worse clinical outcome (*P* = 0.009 and *P* = 0.0008, respectively). However, when these associations were adjusted for the effect of GCS, no difference was detected. As seen in Table 1, no difference in outcome could be detected in a group comparison.

## Discussion

The aim of the current study was to examine the association of CMBs with WM alterations in TBI and their relationship to clinical outcome. This work demonstrates that CMBs in the initial SWI were associated with altered WM microstructure (decreased FA and increased MD) in the follow-up MRI of patients with TBI. However, when adjusting for the clinical severity of TBI, no difference between CMB-positive and CMB-negative patients with TBI in DTI metrics was detected. This is explained by patients with TBI and CMBs having clinically more severe injuries compared with patients with TBI without CMBs. Thus, the effect of CMBs on WM alterations was not independent and was strongly related to clinical severity. This suggests that CMBs and microstructural WM alterations may not be strongly associated aspects of TBI. Also, it is important to note that even though CMBs were associated with more severe injury, a patient with TBI and CMBs can recover with no long-term disability, as well as a patient with TBI without CMBs can show a poor outcome as seen in Table 1.

Our study was one of the few attempts to study the association between diffuse vascular and axonal injury. Similar findings include the work by Andreasen et al., who only found limited colocalization of CMBs in SWI and structural WM disruptions in severe TBI (21). Also, Haber et al. concluded that imaging biomarkers of DAI (FA, MD) and diffuse vascular injury (cerebral blood flow, cerebrovascular reactivity) are spatially independent (22). These prior findings together with current results suggest that substantial WM damage can occur without CMBs. Thus, CMBs have a limited ability to serve as a surrogate for WM damage and highlights the need for development and use of more sensitive methods in detecting DAI. However, our results contradict with the findings by Toth et al., who found a correlation between CMB lesion load in the basal ganglia area and subcortical FA independent of GCS (37). Even though the anatomical segmentation for DTI metrics was far less specific compared to the present study, the more spatially specific localization of CMBs may be more sensitive to associations between CMBs and DTI metrics in related regions. This is supported by Rostowsky et al., who by using tractography detected changes in local WM tract configuration surrounding CMBs seen in SWI in mild TBI (38). Moen et al. found lower FA values in WM regions with CMBs detected with FLAIR and GRE-sequences (39). However, SWI is shown to be a more sensitive method for detecting CMBs (13, 14). Furthermore, Feltrin et al. concluded that CMBs were associated with a subsequent decrease in white matter volume on a whole-brain level, even though they could not predict lower cognitive performance (40).

The three different approaches showed somewhat different results in estimating the associations of CMBs with WM integrity. TBSS showed widespread associations between CMB variables and DTI metrics as well as group differences in DTI metrics between CMB-positive and CMB-negative patients with TBI. Whole brain analysis showed a similar association between CMB variables and DTI metrics, but only found a group difference in FA between the two groups of patients with TBI. The ROI analysis also showed similar results regarding CMB variables but detected no group difference between CMB-positive and negative patients with TBI. This highlights the importance of the decision between different analysis methods for DTI data. Although ROI analysis has been shown to be more sensitive than voxel-wise methods in detecting WM alterations in a certain setting, the use of as many as 48 ROIs and thus having to correct for multiple comparisons decreased the statistical power significantly in this study (10). This may explain why only TBSS was sensitive in detecting the differences between CMB-positive and negative patients with TBI. Some of the regions were also very small in volume, which could be very prone to registration errors. Conflicting results in the region of interest analysis regarding the corticospinal tract and middle cerebellar peduncle may be a manifestation of this.

CMBs were associated with a worse clinical outcome, which supports some earlier evidence (13, 14). However, this contradicts with Tate et al., who suggested that their disagreement can be explained by only including mild TBI whereas earlier work included a wider spectrum considering injury severity (16).

### Limitations

Even though SWI is a sensitive method for detecting CMBs, novel methods and higher field strengths may surpass the sensitivity of the imaging protocol used in the present study (41, 42). The sample size particularly limits the power of the ROI analysis where a multiple comparison correction across 48 anatomical regions was applied. Several inaccuracies in the TBSS pipeline have been reported. Anatomical specificity is one of them, as TBSS may fail to separate adjacent tracts possibly further leading to false significant findings. Also, imperfect alignment can create artificial tubular or sheet-like structures in the FA skeleton that further decrease the anatomical specificity. Additionally, the amount of noise has been shown to affect both the shape of the FA skeleton and the resulting statistical significance (43). Moreover, patients underwent the first MR imaging up to 2 months after injury. This may not be early enough to detect all CMBs as their volume may decrease already in a shorter period of time (15).

## Conclusion

The presence of CMBs was not associated with microstructural WM alterations when adjusting for the clinical severity of TBI. This suggests that WM alterations and CMBs may not be strongly associated aspects in the pathophysiology of TBI.

## Data Availability Statement

The raw data supporting the conclusions of this article will be made available by the authors, without undue reservation.

## Ethics Statement

The studies involving human participants were reviewed and approved by Ethical Review Board of the Hospital District of Southwest Finland. The patients/participants provided their written informed consent to participate in this study.

## Author Contributions

JD: image and statistical analysis, manuscript writing, methodology, and data curation. OT: organizing and planning of the study, interpretation of results, and revision of the manuscript. JP: recruiting patients, data curation, manuscript drafting, and revision of the manuscript. JH: study design and interpretation of MR images. AK: recruiting patients and original TBIcare study design. JF and H-RM: recruiting patients. RT: recruiting patients, collection of data, and revision of the manuscript. EL: statistical analysis. JT: study design and revision of the manuscript. VN: revision of manuscript and original TBIcare study design. DM and PH: original TBIcare study design. MM: data curation, methodology, supervision of image and statistical analyses, drafting the original manuscript, and revision of the manuscript. All authors contributed to the article and approved the submitted version.

## Funding

This work was partially funded by the European Commission under the 7th Framework Programme (FP7-270259-TBIcare), Academy of Finland—Grant # 17379 (JP), Government's Special Financial Transfer tied to academic research in Health Sciences (Finland) (OT, JP), the Maire Taponen foundation (JP), University of Turku Graduate School funding (MM), the Royal College of Surgeons (PH), NIHR Research Professorship and the NIHR Cambridge BRC (PH), NIHR Research UK (through a Senior Investigator Award and the Cambridge Biomedical Research Center) (DM), Academy of Medical Sciences/The Health Foundation Clinician Scientist Fellowship (VN).

## Conflict of Interest

VN reports a grant from Roche Pharmaceuticals, outside the submitted work. DM reports grants from GlaxoSmithKline and personal fees from NeuroTraumaSciences, Pfizer, Calico, PressuraNeuro, Lantmannen, Integra Neurosciences, Gryphon, and Cortirio, outside the submitted work. JP has received a speaker's free from the Finnish Medical Association. The remaining authors declare that the research was conducted in the absence of any commercial or financial relationships that could be construed as a potential conflict of interest.

## Publisher's Note

All claims expressed in this article are solely those of the authors and do not necessarily represent those of their affiliated organizations, or those of the publisher, the editors and the reviewers. Any product that may be evaluated in this article, or claim that may be made by its manufacturer, is not guaranteed or endorsed by the publisher.

## Abbreviations

DAI: diffuse axonal injury
TBI: traumatic brain injury
WM: white matter
DTI: diffusion tensor imaging
CMB: cerebral microbleed
SWI: susceptibility-weighted imaging
GOSe: Glasgow Outcome Scale extended
GCS: Glasgow Coma Scale
PTA: post-traumatic amnesia
FA: fractional anisotropy
MD: mean diffusivity
DW-MRI: diffusion-weighted magnetic resonance imaging
ROI: region of interest
TBSS: tract-based spatial statistics
TBIcare: EU-funded International Evidence-based Diagnostic and Treatment Planning Solution for Traumatic Brain Injuries
FSL: Oxford Center for Functional MRI of the Brain Software Library.
