# Supplementary material for: Cerebral Microbleeds and Structural White Matter Integrity in Patients With Traumatic Brain Injury—A Diffusion Tensor Imaging Study
Source: Front Neurol. 2022 May 31;13:888815. doi: 10.3389/fneur.2022.888815 (PMC9194845; doi:10.3389/fneur.2022.888815)
Supplement: Supplementary file 1 [file Table_1.DOCX]

| **Supplementary material.** Statistically significant associations and group comparisons between cerebral microbleeds (CMB) and white matter microstructural properties in the region of interest analysis. Non-significant associations are only shown for group comparisons between CMB-positive and CMB-negative patients with TBI. All reported P-values are adjusted for age, sex, education level, and time from injury to diffusion tensor imaging. All P-values are corrected for multiple comparisons for 48 anatomical regions using false discovery rate unless specified separately. Mean diffusivity (MD) values are presented in the magnitude of 10^-3^. FA = fractional anisotropy. | | | | | | | |
| --- | --- | --- | --- | --- | --- | --- | --- |
| **CMB grading vs FA** | | | | | |  |  |
| Region | | | | | Association | P-value | P-value adjusted for injury severity |
| Anterior corona radiata L | | | | | negative | 0.023 | 0.974 |
| Anterior corona radiata R | | | | | negative | 0.025 | 0.974 |
| Anterior limb of internal capsule L | | | | | negative | 0.021 | 0.974 |
| Anterior limb of internal capsule R | | | | | negative | 0.006 | 0.974 |
| Body of corpus callosum | | | | | negative | <0.001 | 0.974 |
| Cerebral peduncle L | | | | | negative | 0.002 | 0.974 |
| Cerebral peduncle R | | | | | negative | 0.003 | 0.974 |
| Cingulum cingulate gyrus L | | | | | negative | 0.004 | 0.974 |
| Cingulum cingulate gyrus R | | | | | negative | 0.042 | 0.974 |
| Cingulum hippocampus L | | | | | negative | 0.048 | 0.974 |
| Corticospinal tract L | | | | | negative | 0.016 | 0.974 |
| Corticospinal tract R | | | | | negative | 0.008 | 0.974 |
| External capsule L | | | | | negative | 0.018 | 0.974 |
| External capsule R | | | | | negative | 0.004 | 0.974 |
| Fornix column and body of fornix | | | | | negative | 0.008 | 0.974 |
| Fornix cres/Stria terminalis L | | | | | negative | <0.001 | 0.974 |
| Fornix cres/Stria terminalis R | | | | | negative | 0.002 | 0.974 |
| Genu of corpus callosum | | | | | negative | 0.007 | 0.974 |
| Medial lemniscus L | | | | | negative | 0.016 | 0.974 |
| Medial lemniscus R | | | | | negative | 0.005 | 0.974 |
| Middle cerebellar peduncle | | | | | negative | 0.008 | 0.974 |
| Pontine crossing tract a part of MCP | | | | | negative | 0.010 | 0.974 |
| Posterior corona radiata L | | | | | negative | <0.001 | 0.974 |
| Posterior corona radiata R | | | | | negative | 0.002 | 0.974 |
| Posterior limb of internal capsule L | | | | | negative | 0.008 | 0.974 |
| Posterior limb of internal capsule R | | | | | negative | 0.002 | 0.974 |
| Posterior thalamic radiation including optic radiation L | | | | | negative | 0.002 | 0.974 |
| Posterior thalamic radiation including optic radiation R | | | | | negative | 0.002 | 0.974 |
| Retrolenticular part of internal capsule L | | | | | negative | 0.002 | 0.974 |
| Retrolenticular part of internal capsule R | | | | | negative | 0.003 | 0.974 |
| Sagittal stratum including inferior longitudinal fasciculus and inferior fronto-occipital fasciculus L | | | | | negative | <0.001 | 0.974 |
| Sagittal stratum including inferior longitudinal fasciculus and inferior fronto-occipital fasciculus R | | | | | negative | <0.001 | 0.974 |
| Splenium of corpus callosum | | | | | negative | 0.001 | 0.974 |
| Superior cerebellar peduncle L | | | | | negative | 0.002 | 0.974 |
| Superior cerebellar peduncle R | | | | | negative | 0.004 | 0.974 |
| Superior corona radiata L | | | | | negative | 0.005 | 0.974 |
| Superior corona radiata R | | | | | negative | 0.002 | 0.974 |
| Superior fronto-occipital fasciculus, could be a part of anterior internal capsule L | | | | | negative | 0.048 | 0.974 |
| Superior fronto-occipital fasciculus, could be a part of anterior internal capsule R | | | | | negative | 0.005 | 0.974 |
| Superior longitudinal fasciculus L | | | | | negative | 0.002 | 0.974 |
| Superior longitudinal fasciculus R | | | | | negative | 0.002 | 0.974 |
| Tapetum L | | | | | negative | 0.005 | 0.974 |
| Tapetum R | | | | | negative | 0.027 | 0.974 |
| Uncinate fasciculus L | | | | | negative | 0.029 | 0.974 |
| Uncinate fasciculus R | | | | | negative | 0.005 | 0.974 |
| **CMB grading vs MD** | | | | |  |  |  |
| Region | | | | | Association | P-value | P-value adjusted for injury severity |
| Anterior corona radiata R | | | | | positive | 0.022 | 0.970 |
| Body of corpus callosum | | | | | positive | 0.003 | 0.970 |
| Cingulum cingulate gyrus R | | | | | positive | 0.031 | 0.993 |
| Cingulum hippocampus L | | | | | positive | 0.025 | 0.993 |
| Cingulum hippocampus R | | | | | positive | 0.031 | 0.988 |
| Corticospinal tract L | | | | | positive | 0.039 | 0.993 |
| Corticospinal tract R | | | | | positive | 0.045 | 0.988 |
| External capsule R | | | | | positive | 0.045 | 0.970 |
| Fornix column and body of fornix | | | | | positive | 0.045 | 0.970 |
| Fornix cres/Stria terminalis L | | | | | positive | 0.003 | 0.970 |
| Fornix cres/Stria terminalis R | | | | | positive | 0.003 | 0.988 |
| Inferior cerebellar peduncle L | | | | | positive | 0.028 | 0.970 |
| Inferior cerebellar peduncle R | | | | | positive | 0.025 | 0.970 |
| Middle cerebellar peduncle | | | | | positive | 0.003 | 0.970 |
| Posterior corona radiata L | | | | | positive | 0.004 | 0.970 |
| Posterior corona radiata R | | | | | positive | 0.003 | 0.970 |
| Posterior limb of internal capsule R | | | | | positive | 0.017 | 0.970 |
| Posterior thalamic radiation including optic radiation R | | | | | positive | 0.019 | 0.970 |
| Retrolenticular part of internal capsule L | | | | | positive | 0.003 | 0.970 |
| Retrolenticular part of internal capsule R | | | | | positive | 0.003 | 0.970 |
| Sagittal stratum including inferior longitudinal fasciculus and inferior fronto-occipital fasciculus L | | | | | positive | 0.003 | 0.970 |
| Sagittal stratum including inferior longitudinal fasciculus and inferior fronto-occipital fasciculus R | | | | | positive | 0.003 | 0.970 |
| Superior cerebellar peduncle L | | | | | positive | 0.005 | 0.970 |
| Superior cerebellar peduncle R | | | | | positive | 0.003 | 0.970 |
| Superior corona radiata L | | | | | positive | 0.005 | 0.970 |
| Superior corona radiata R | | | | | positive | 0.003 | 0.970 |
| Superior fronto-occipital fasciculus, could be a part of anterior internal capsule L | | | | | positive | 0.039 | 0.970 |
| Superior fronto-occipital fasciculus, could be a part of anterior internal capsule R | | | | | positive | 0.031 | 0.970 |
| Superior longitudinal fasciculus L | | | | | positive | 0.036 | 0.988 |
| Superior longitudinal fasciculus R | | | | | positive | 0.044 | 0.993 |
| **Total lesion load vs FA** | | | | |  |  |  |
| Region | | | | | Association | P-value | P-value adjusted for injury severity |
| Anterior corona radiata L | | | | | negative | 0.007 | 0.998 |
| Anterior corona radiata R | | | | | negative | 0.014 | 0.998 |
| Anterior limb of internal capsule L | | | | | negative | 0.009 | 0.998 |
| Anterior limb of internal capsule R | | | | | negative | 0.006 | 0.998 |
| Body of corpus callosum | | | | | negative | <0.001 | 0.998 |
| Cerebral peduncle L | | | | | negative | 0.001 | 0.998 |
| Cerebral peduncle R | | | | | negative | 0.006 | 0.998 |
| Cingulum cingulate gyrus L | | | | | negative | 0.007 | 0.998 |
| Cingulum cingulate gyrus R | | | | | negative | 0.014 | 0.998 |
| Corticospinal tract L | | | | | negative | 0.025 | 0.998 |
| Corticospinal tract R | | | | | negative | 0.028 | 0.998 |
| External capsule R | | | | | negative | 0.025 | 0.998 |
| Fornix column and body of fornix | | | | | negative | 0.007 | 0.998 |
| Fornix cres/Stria terminalis L | | | | | negative | 0.001 | 0.998 |
| Fornix cres/Stria terminalis R | | | | | negative | 0.007 | 0.998 |
| Genu of corpus callosum | | | | | negative | 0.006 | 0.998 |
| Medial lemniscus L | | | | | negative | 0.037 | 0.998 |
| Medial lemniscus R | | | | | negative | 0.012 | 0.998 |
| Middle cerebellar peduncle | | | | | negative | 0.020 | 0.998 |
| Posterior corona radiata L | | | | | negative | <0.001 | 0.998 |
| Posterior corona radiata R | | | | | negative | 0.003 | 0.998 |
| Posterior limb of internal capsule L | | | | | negative | 0.009 | 0.998 |
| Posterior limb of internal capsule R | | | | | negative | 0.006 | 0.998 |
| Posterior thalamic radiation including optic radiation L | | | | | negative | 0.002 | 0.998 |
| Posterior thalamic radiation including optic radiation R | | | | | negative | 0.005 | 0.998 |
| Retrolenticular part of internal capsule L | | | | | negative | 0.007 | 0.998 |
| Retrolenticular part of internal capsule R | | | | | negative | 0.005 | 0.998 |
| Sagittal stratum including inferior longitudinal fasciculus and inferior fronto-occipital fasciculus L | | | | | negative | 0.001 | 0.998 |
| Sagittal stratum including inferior longitudinal fasciculus and inferior fronto-occipital fasciculus R | | | | | negative | 0.002 | 0.998 |
| Splenium of corpus callosum | | | | | negative | 0.001 | 0.998 |
| Superior cerebellar peduncle L | | | | | negative | 0.005 | 0.998 |
| Superior cerebellar peduncle R | | | | | negative | 0.007 | 0.998 |
| Superior corona radiata L | | | | | negative | 0.013 | 0.998 |
| Superior corona radiata R | | | | | negative | 0.005 | 0.998 |
| Superior fronto-occipital fasciculus, could be a part of anterior internal capsule R | | | | | negative | 0.006 | 0.998 |
| Superior longitudinal fasciculus L | | | | | negative | 0.001 | 0.998 |
| Superior longitudinal fasciculus R | | | | | negative | 0.007 | 0.998 |
| Tapetum L | | | | | negative | 0.007 | 0.998 |
| Tapetum R | | | | | negative | 0.042 | 0.998 |
| Uncinate fasciculus L | | | | | negative | 0.038 | 0.998 |
| Uncinate fasciculus R | | | | | negative | 0.025 | 0.998 |
| **Total lesion load vs MD** | | | | |  |  |  |
| Region | | | | | Association | P-value | P-value adjusted for injury severity |
| Anterior corona radiata L | | | | | positive | 0.026 | 0.889 |
| Anterior corona radiata R | | | | | positive | 0.022 | 0.990 |
| Body of corpus callosum | | | | | positive | 0.004 | 0.990 |
| Cingulum cingulate gyrus L | | | | | positive | 0.017 | 0.596 |
| Cingulum cingulate gyrus R | | | | | positive | 0.004 | 0.889 |
| Cingulum hippocampus L | | | | | positive | 0.016 | 0.990 |
| Cingulum hippocampus R | | | | | positive | 0.025 | 0.990 |
| Corticospinal tract L | | | | | positive | 0.017 | 0.990 |
| Corticospinal tract R | | | | | positive | 0.032 | 0.990 |
| External capsule L | | | | | positive | 0.046 | 0.990 |
| External capsule R | | | | | positive | 0.045 | 0.990 |
| Fornix column and body of fornix | | | | | positive | 0.036 | 0.889 |
| Fornix cres/Stria terminalis L | | | | | positive | <0.001 | 0.877 |
| Fornix cres/Stria terminalis R | | | | | positive | 0.004 | 0.990 |
| Genu of corpus callosum | | | | | positive | 0.048 | 0.990 |
| Inferior cerebellar peduncle L | | | | | positive | 0.028 | 0.877 |
| Inferior cerebellar peduncle R | | | | | positive | 0.028 | 0.990 |
| Middle cerebellar peduncle | | | | | positive | 0.009 | 0.990 |
| Posterior corona radiata L | | | | | positive | <0.001 | 0.596 |
| Posterior corona radiata R | | | | | positive | <0.001 | 0.877 |
| Posterior limb of internal capsule R | | | | | positive | 0.014 | 0.990 |
| Retrolenticular part of internal capsule L | | | | | positive | 0.004 | 0.990 |
| Retrolenticular part of internal capsule R | | | | | positive | 0.004 | 0.990 |
| Sagittal stratum including inferior longitudinal fasciculus and inferior fronto-occipital fasciculus L | | | | | positive | 0.003 | 0.889 |
| Sagittal stratum including inferior longitudinal fasciculus and inferior fronto-occipital fasciculus R | | | | | positive | 0.001 | 0.990 |
| Superior cerebellar peduncle L | | | | | positive | 0.017 | 0.990 |
| Superior cerebellar peduncle R | | | | | positive | 0.013 | 0.990 |
| Superior corona radiata L | | | | | positive | <0.001 | 0.596 |
| Superior corona radiata R | | | | | positive | <0.001 | 0.877 |
| Superior fronto-occipital fasciculus, could be a part of anterior internal capsule L | | | | | positive | 0.043 | 0.990 |
| Superior fronto-occipital fasciculus, could be a part of anterior internal capsule R | | | | | positive | 0.025 | 0.954 |
| Superior longitudinal fasciculus L | | | | | positive | 0.004 | 0.678 |
| Superior longitudinal fasciculus R | | | | | positive | 0.004 | 0.877 |
| Uncinate fasciculus L | | | | | positive | 0.029 | 0.889 |
| **FA** | **CMB-positive TBI** | | **CMB-negative TBI** | |  |  |  |
| Region | FA | (sd) | FA | (sd) | P-value without multiple comparison adjustment | P-value adjusted for multiple comparisons | P-value adjusted for multiple comparisons and injury severity |
| Anterior corona radiata L | 0.419 | 0.036 | 0.429 | 0.029 | 0.208 | 0.262 | 0.997 |
| Anterior corona radiata R | 0.412 | 0.028 | 0.423 | 0.030 | 0.172 | 0.231 | 0.997 |
| Anterior limb of internal capsule L | 0.514 | 0.029 | 0.522 | 0.024 | 0.403 | 0.440 | 0.997 |
| Anterior limb of internal capsule R | 0.511 | 0.032 | 0.523 | 0.026 | 0.213 | 0.262 | 0.997 |
| Body of corpus callosum | 0.568 | 0.056 | 0.606 | 0.028 | 0.006 | 0.096 | 0.997 |
| Cerebral peduncle L | 0.597 | 0.038 | 0.618 | 0.028 | 0.066 | 0.148 | 0.997 |
| Cerebral peduncle R | 0.594 | 0.052 | 0.617 | 0.027 | 0.069 | 0.148 | 0.997 |
| Cingulum cingulate gyrus L | 0.481 | 0.036 | 0.497 | 0.031 | 0.095 | 0.163 | 0.997 |
| Cingulum cingulate gyrus R | 0.437 | 0.040 | 0.444 | 0.030 | 0.594 | 0.633 | 0.997 |
| Cingulum hippocampus L | 0.376 | 0.040 | 0.381 | 0.034 | 0.705 | 0.720 | 0.997 |
| Cingulum hippocampus R | 0.378 | 0.040 | 0.384 | 0.033 | 0.610 | 0.636 | 0.997 |
| Corticospinal tract L | 0.448 | 0.040 | 0.465 | 0.034 | 0.264 | 0.309 | 0.997 |
| Corticospinal tract R | 0.443 | 0.049 | 0.464 | 0.034 | 0.161 | 0.231 | 0.997 |
| External capsule L | 0.405 | 0.022 | 0.415 | 0.016 | 0.123 | 0.197 | 0.997 |
| External capsule R | 0.391 | 0.020 | 0.402 | 0.015 | 0.047 | 0.140 | 0.997 |
| Fornix column and body of fornix | 0.340 | 0.077 | 0.372 | 0.063 | 0.091 | 0.161 | 0.997 |
| Fornix cres / Stria terminalis cannot be resolved with current resolution L | 0.470 | 0.052 | 0.501 | 0.030 | 0.010 | 0.096 | 0.997 |
| Fornix cres / Stria terminalis cannot be resolved with current resolution R | 0.441 | 0.055 | 0.465 | 0.036 | 0.086 | 0.161 | 0.997 |
| Genu of corpus callosum | 0.526 | 0.037 | 0.537 | 0.024 | 0.173 | 0.231 | 0.997 |
| Inferior cerebellar peduncle L | 0.451 | 0.023 | 0.458 | 0.025 | 0.378 | 0.422 | 0.997 |
| Inferior cerebellar peduncle R | 0.448 | 0.024 | 0.457 | 0.030 | 0.104 | 0.173 | 0.997 |
| Medial lemniscus L | 0.555 | 0.030 | 0.568 | 0.026 | 0.167 | 0.231 | 0.997 |
| Medial lemniscus R | 0.548 | 0.027 | 0.564 | 0.025 | 0.068 | 0.148 | 0.997 |
| Middle cerebellar peduncle | 0.414 | 0.023 | 0.427 | 0.020 | 0.087 | 0.161 | 0.997 |
| Pontine crossing tract a part of MCP | 0.421 | 0.030 | 0.442 | 0.030 | 0.043 | 0.140 | 0.997 |
| Posterior corona radiata L | 0.440 | 0.041 | 0.471 | 0.028 | 0.006 | 0.096 | 0.997 |
| Posterior corona radiata R | 0.453 | 0.036 | 0.478 | 0.030 | 0.024 | 0.128 | 0.997 |
| Posterior limb of internal capsule L | 0.595 | 0.029 | 0.606 | 0.018 | 0.193 | 0.251 | 0.997 |
| Posterior limb of internal capsule R | 0.586 | 0.036 | 0.602 | 0.018 | 0.066 | 0.148 | 0.997 |
| Posterior thalamic radiation include optic radiation L | 0.534 | 0.048 | 0.561 | 0.030 | 0.032 | 0.129 | 0.997 |
| Posterior thalamic radiation include optic radiation R | 0.543 | 0.053 | 0.570 | 0.033 | 0.051 | 0.140 | 0.997 |
| Retrolenticular part of internal capsule L | 0.554 | 0.039 | 0.578 | 0.023 | 0.005 | 0.096 | 0.997 |
| Retrolenticular part of internal capsule R | 0.529 | 0.039 | 0.551 | 0.027 | 0.024 | 0.128 | 0.997 |
| Sagittal stratum include inferior longitidinal fasciculus and inferior fronto-occipital fasciculus L | 0.455 | 0.037 | 0.481 | 0.025 | 0.010 | 0.096 | 0.997 |
| Sagittal stratum include inferior longitidinal fasciculus and inferior fronto-occipital fasciculus R | 0.482 | 0.041 | 0.511 | 0.030 | 0.014 | 0.109 | 0.997 |
| Splenium of corpus callosum | 0.666 | 0.050 | 0.692 | 0.025 | 0.018 | 0.124 | 1.000 |
| Superior cerebellar peduncle L | 0.505 | 0.037 | 0.531 | 0.029 | 0.052 | 0.140 | 0.997 |
| Superior cerebellar peduncle R | 0.529 | 0.046 | 0.554 | 0.032 | 0.130 | 0.201 | 0.997 |
| Superior corona radiata L | 0.470 | 0.034 | 0.483 | 0.018 | 0.090 | 0.161 | 0.997 |
| Superior corona radiata R | 0.461 | 0.028 | 0.480 | 0.020 | 0.027 | 0.128 | 0.997 |
| Superior fronto-occipital fasciculus could be a part of anterior internal capsule L | 0.454 | 0.042 | 0.459 | 0.028 | 0.756 | 0.756 | 0.997 |
| Superior fronto-occipital fasciculus could be a part of anterior internal capsule R | 0.458 | 0.037 | 0.476 | 0.027 | 0.050 | 0.140 | 0.997 |
| Superior longitudinal fasciculus L | 0.464 | 0.027 | 0.479 | 0.021 | 0.032 | 0.129 | 1.000 |
| Superior longitudinal fasciculus R | 0.464 | 0.025 | 0.476 | 0.019 | 0.071 | 0.148 | 0.997 |
| Tapetum L | 0.267 | 0.053 | 0.295 | 0.031 | 0.052 | 0.140 | 0.997 |
| Tapetum R | 0.370 | 0.057 | 0.390 | 0.035 | 0.295 | 0.337 | 0.997 |
| Uncinate fasciculus L | 0.426 | 0.038 | 0.443 | 0.033 | 0.235 | 0.282 | 0.997 |
| Uncinate fasciculus R | 0.439 | 0.041 | 0.457 | 0.031 | 0.150 | 0.226 | 0.997 |
| **MD** | **CMB-positive TBI** | | **CMB-negative TBI** | |  |  |  |
| Region | MD | (sd) | MD | (sd) | P-value without multiple comparison adjustment | P-value adjusted for multiple comparisons | P-value adjusted for multiple comparisons and injury severity |
| Anterior corona radiata L | 0.787 | (0.043) | 0.773 | (0.044) | 0.272 | 0.409 | 0.993 |
| Anterior corona radiata R | 0.808 | (0.050) | 0.781 | (0.045) | 0.058 | 0.197 | 0.951 |
| Anterior limb of internal capsule L | 0.730 | (0.018) | 0.727 | (0.029) | 0.572 | 0.624 | 0.951 |
| Anterior limb of internal capsule R | 0.735 | (0.021) | 0.731 | (0.028) | 0.540 | 0.624 | 0.951 |
| Body of corpus callosum | 0.927 | (0.053) | 0.891 | (0.040) | 0.038 | 0.163 | 0.951 |
| Cerebral peduncle L | 0.800 | (0.035) | 0.796 | (0.028) | 0.663 | 0.677 | 0.993 |
| Cerebral peduncle R | 0.822 | (0.031) | 0.815 | (0.035) | 0.355 | 0.448 | 0.951 |
| Cingulum cingulate gyrus L | 0.745 | (0.026) | 0.738 | (0.029) | 0.321 | 0.448 | 0.951 |
| Cingulum cingulate gyrus R | 0.761 | (0.034) | 0.751 | (0.026) | 0.196 | 0.376 | 0.951 |
| Cingulum hippocampus L | 0.892 | (0.065) | 0.868 | (0.083) | 0.208 | 0.376 | 0.951 |
| Cingulum hippocampus R | 0.869 | (0.075) | 0.844 | (0.059) | 0.235 | 0.376 | 0.951 |
| Corticospinal tract L | 0.876 | (0.052) | 0.856 | (0.043) | 0.343 | 0.448 | 0.951 |
| Corticospinal tract R | 0.890 | (0.061) | 0.860 | (0.045) | 0.128 | 0.266 | 0.951 |
| External capsule L | 0.752 | (0.018) | 0.745 | (0.028) | 0.219 | 0.376 | 0.951 |
| External capsule R | 0.764 | (0.021) | 0.754 | (0.028) | 0.087 | 0.231 | 0.951 |
| Fornix column and body of fornix | 1.710 | (0.244) | 1.643 | (0.227) | 0.229 | 0.376 | 0.951 |
| Fornix cres / Stria terminalis cannot be resolved with current resolution L | 0.933 | (0.068) | 0.889 | (0.056) | 0.029 | 0.163 | 0.951 |
| Fornix cres / Stria terminalis cannot be resolved with current resolution R | 1.040 | (0.092) | 0.997 | (0.074) | 0.107 | 0.244 | 0.951 |
| Genu of corpus callosum | 1.117 | (0.075) | 1.089 | (0.083) | 0.351 | 0.448 | 0.951 |
| Inferior cerebellar peduncle L | 0.930 | (0.081) | 0.908 | (0.083) | 0.448 | 0.551 | 0.951 |
| Inferior cerebellar peduncle R | 0.923 | (0.068) | 0.889 | (0.075) | 0.082 | 0.231 | 0.951 |
| Medial lemniscus L | 0.771 | (0.038) | 0.757 | (0.028) | 0.229 | 0.376 | 0.951 |
| Medial lemniscus R | 0.784 | (0.044) | 0.763 | (0.035) | 0.066 | 0.198 | 0.951 |
| Middle cerebellar peduncle | 0.694 | (0.029) | 0.674 | (0.022) | 0.004 | 0.160 | 0.951 |
| Pontine crossing tract a part of MCP | 0.751 | (0.051) | 0.741 | (0.041) | 0.608 | 0.643 | 0.951 |
| Posterior corona radiata L | 0.837 | (0.055) | 0.801 | (0.045) | 0.031 | 0.163 | 0.951 |
| Posterior corona radiata R | 0.839 | (0.055) | 0.809 | (0.043) | 0.044 | 0.163 | 0.951 |
| Posterior limb of internal capsule L | 0.706 | (0.027) | 0.701 | (0.022) | 0.616 | 0.643 | 0.951 |
| Posterior limb of internal capsule R | 0.726 | (0.033) | 0.712 | (0.025) | 0.099 | 0.237 | 0.951 |
| Posterior thalamic radiation include optic radiation L | 0.938 | (0.072) | 0.920 | (0.086) | 0.734 | 0.734 | 0.951 |
| Posterior thalamic radiation include optic radiation R | 0.892 | (0.045) | 0.860 | (0.044) | 0.041 | 0.163 | 0.951 |
| Retrolenticular part of internal capsule L | 0.787 | (0.043) | 0.759 | (0.038) | 0.020 | 0.160 | 0.951 |
| Retrolenticular part of internal capsule R | 0.817 | (0.048) | 0.789 | (0.034) | 0.014 | 0.160 | 0.951 |
| Sagittal stratum include inferior longitidinal fasciculus and inferior fronto-occipital fasciculus L | 0.935 | (0.066) | 0.896 | (0.067) | 0.020 | 0.160 | 0.951 |
| Sagittal stratum include inferior longitidinal fasciculus and inferior fronto-occipital fasciculus R | 0.893 | (0.050) | 0.855 | (0.051) | 0.015 | 0.160 | 0.951 |
| Splenium of corpus callosum | 0.830 | (0.032) | 0.822 | (0.037) | 0.569 | 0.624 | 0.951 |
| Superior cerebellar peduncle L | 1.199 | (0.093) | 1.140 | (0.081) | 0.012 | 0.160 | 0.951 |
| Superior cerebellar peduncle R | 1.091 | (0.082) | 1.051 | (0.062) | 0.036 | 0.163 | 0.951 |
| Superior corona radiata L | 0.722 | (0.034) | 0.704 | (0.029) | 0.063 | 0.198 | 0.951 |
| Superior corona radiata R | 0.725 | (0.039) | 0.704 | (0.028) | 0.044 | 0.163 | 0.951 |
| Superior fronto-occipital fasciculus could be a part of anterior internal capsule L | 0.734 | (0.066) | 0.715 | (0.072) | 0.326 | 0.448 | 0.951 |
| Superior fronto-occipital fasciculus could be a part of anterior internal capsule R | 0.723 | (0.065) | 0.700 | (0.038) | 0.114 | 0.249 | 0.951 |
| Superior longitudinal fasciculus L | 0.718 | (0.024) | 0.708 | (0.026) | 0.093 | 0.234 | 0.951 |
| Superior longitudinal fasciculus R | 0.735 | (0.027) | 0.728 | (0.024) | 0.270 | 0.409 | 0.951 |
| Tapetum L | 2.093 | (0.225) | 2.053 | (0.155) | 0.556 | 0.624 | 0.951 |
| Tapetum R | 1.698 | (0.171) | 1.634 | (0.139) | 0.216 | 0.376 | 0.951 |
| Uncinate fasciculus L | 0.763 | (0.027) | 0.759 | (0.043) | 0.329 | 0.448 | 0.951 |
| Uncinate fasciculus R | 0.775 | (0.031) | 0.768 | (0.034) | 0.540 | 0.624 | 0.951 |
| **FA** | | | **CMB-positive TBI,** | | **Controls,** |  |  |
| Region | | | FA | (sd) | FA | (sd) | P-value |
| Anterior corona radiata L | | | 0.419 | (0.036) | 0.446 | (0.029) | 0.047 |
| Anterior corona radiata R | | | 0.412 | (0.028) | 0.446 | (0.030) | 0.030 |
| Anterior limb of internal capsule L | | | 0.514 | (0.029) | 0.536 | (0.024) | 0.047 |
| Body of corpus callosum | | | 0.568 | (0.056) | 0.623 | (0.028) | 0.024 |
| Cerebral peduncle L | | | 0.597 | (0.038) | 0.627 | (0.028) | 0.031 |
| Cerebral peduncle R | | | 0.594 | (0.052) | 0.633 | (0.027) | 0.045 |
| Cingulum cingulate gyrus L | | | 0.481 | (0.036) | 0.512 | (0.031) | 0.030 |
| Cingulum cingulate gyrus R | | | 0.437 | (0.040) | 0.468 | (0.030) | 0.047 |
| Corticospinal tract L | | | 0.448 | (0.040) | 0.487 | (0.034) | 0.022 |
| Corticospinal tract R | | | 0.443 | (0.049) | 0.476 | (0.034) | 0.047 |
| External capsule L | | | 0.405 | (0.022) | 0.425 | (0.016) | 0.044 |
| External capsule R | | | 0.391 | (0.020) | 0.408 | (0.015) | 0.040 |
| Fornix column and body of fornix | | | 0.340 | (0.077) | 0.422 | (0.063) | 0.030 |
| Fornix cres/Stria terminalis L | | | 0.470 | (0.052) | 0.519 | (0.030) | 0.040 |
| Genu of corpus callosum | | | 0.526 | (0.037) | 0.552 | (0.024) | 0.047 |
| Medial lemniscus R | | | 0.548 | (0.027) | 0.569 | (0.025) | 0.041 |
| Middle cerebellar peduncle | | | 0.414 | (0.023) | 0.460 | (0.020) | <0.001 |
| Pontine crossing tract a part of MCP | | | 0.421 | (0.030) | 0.453 | (0.030) | 0.030 |
| Posterior corona radiata L | | | 0.440 | (0.041) | 0.482 | (0.028) | 0.024 |
| Posterior corona radiata R | | | 0.453 | (0.036) | 0.492 | (0.030) | 0.024 |
| Posterior limb of internal capsule R | | | 0.586 | (0.036) | 0.613 | (0.018) | 0.043 |
| Retrolenticular part of internal capsule L | | | 0.554 | (0.039) | 0.585 | (0.023) | 0.044 |
| Retrolenticular part of internal capsule R | | | 0.529 | (0.039) | 0.559 | (0.027) | 0.042 |
| Sagittal stratum including inferior longitudinal fasciculus and inferior fronto-occipital fasciculus L | | | 0.455 | (0.037) | 0.485 | (0.025) | 0.047 |
| Sagittal stratum including inferior longitudinal fasciculus and inferior fronto-occipital fasciculus R | | | 0.482 | (0.041) | 0.514 | (0.030) | 0.042 |
| Superior corona radiata L | | | 0.470 | (0.034) | 0.498 | (0.018) | 0.039 |
| Superior corona radiata R | | | 0.461 | (0.028) | 0.494 | (0.020) | 0.012 |
| Superior fronto-occipital fasciculus, could be a part of anterior internal capsule R | | | 0.458 | (0.037) | 0.489 | (0.027) | 0.040 |
| **MD** | | | **CMB-positive TBI,** | | **Controls,** |  |  |
| Region | | | MD | (sd) | MD | (sd) | P-value |
| Anterior corona radiata L | | | 0.787 | (0.043) | 0.750 | (0.044) | 0.040 |
| Anterior corona radiata R | | | 0.808 | (0.050) | 0.754 | (0.045) | 0.025 |
| Anterior limb of internal capsule L | | | 0.730 | (0.018) | 0.692 | (0.029) | 0.019 |
| Anterior limb of internal capsule R | | | 0.735 | (0.021) | 0.707 | (0.028) | 0.030 |
| Body of corpus callosum | | | 0.927 | (0.053) | 0.867 | (0.040) | 0.021 |
| Cingulum hippocampus L | | | 0.892 | (0.065) | 0.821 | (0.083) | 0.028 |
| Cingulum hippocampus R | | | 0.869 | (0.075) | 0.799 | (0.059) | 0.030 |
| External capsule R | | | 0.764 | (0.021) | 0.735 | (0.028) | 0.035 |
| Fornix cres/Stria terminalis L | | | 0.933 | (0.068) | 0.839 | (0.056) | 0.019 |
| Fornix cres/Stria terminalis R | | | 1.040 | (0.092) | 0.943 | (0.074) | 0.022 |
| Middle cerebellar peduncle | | | 0.694 | (0.029) | 0.750 | (0.022) | 0.009 |
| Posterior corona radiata L | | | 0.837 | (0.055) | 0.775 | (0.045) | 0.021 |
| Posterior corona radiata R | | | 0.839 | (0.055) | 0.788 | (0.043) | 0.030 |
| Posterior limb of internal capsule L | | | 0.706 | (0.027) | 0.678 | (0.022) | 0.028 |
| Posterior limb of internal capsule R | | | 0.726 | (0.033) | 0.690 | (0.025) | 0.022 |
| Posterior thalamic radiation including optic radiation R | | | 0.892 | (0.045) | 0.840 | (0.044) | 0.035 |
| Retrolenticular part of internal capsule L | | | 0.787 | (0.043) | 0.737 | (0.038) | 0.022 |
| Retrolenticular part of internal capsule R | | | 0.817 | (0.048) | 0.758 | (0.034) | 0.019 |
| Sagittal stratum including inferior longitudinal fasciculus and inferior fronto-occipital fasciculus R | | | 0.893 | (0.050) | 0.832 | (0.051) | 0.022 |
| Superior corona radiata L | | | 0.722 | (0.034) | 0.679 | (0.029) | 0.019 |
| Superior corona radiata R | | | 0.725 | (0.039) | 0.685 | (0.028) | 0.021 |
| Superior fronto-occipital fasciculus, could be a part of anterior internal capsule L | | | 0.734 | (0.066) | 0.671 | (0.072) | 0.045 |
| Uncinate fasciculus L | | | 0.763 | (0.027) | 0.718 | (0.043) | 0.019 |
| Uncinate fasciculus R | | | 0.775 | (0.031) | 0.739 | (0.034) | 0.039 |
| **FA** | | | **CMB-negative TBI,** | | **Controls,** |  |  |
| Region | | | FA | (sd) | FA | (sd) | P-value |
| Middle cerebellar peduncle | | | 0.427 | (0.023) | 0.46 | (0.02) | <0.001 |
| **MD** | | | **CMB-negative TBI,** | | **Controls,** |  |  |
| Region | | | MD | (sd) | MD | (sd) | P-value |
| Corticospinal tract L | | | 0.856 | (0.052) | 0.92 | (0.043) | 0.014 |
| Middle cerebellar peduncle | | | 0.674 | (0.029) | 0.75 | (0.022) | <0.001 |
